# Supplementary material for: MRI Apparent Diffusion Coefficient (ADC) as a Biomarker of Tumour Response: Imaging-Pathology Correlation in Patients with Hepatic Metastases from Colorectal Cancer (EORTC 1423)
Source: Cancers (Basel). 2023 Jul 12;15(14):3580. doi: 10.3390/cancers15143580 (PMC10377224; doi:10.3390/cancers15143580)
Supplement: Supplementary file 1 [file cancers-15-03580-s001.zip › cancers-2422900-supplementary.pdf]

Supplementary material

Phantom acquisition and quality control procedures

The phantom consisted of an acrylic container containing 5 vials of polyvinylpyrrolidone (PVP) solutions of different concentrations, ranging from 0% (distilled water) to 25% w/w, to create a range of different MR contrast parameters. the apparent diffusion coefficient (ADC) at 0° C ranged from (1. 12 +/- 0. 01) x $10^{-3}mm^2/s$ for pure water to (0. 48 +/- 0. 02) X $10^{-3}mm^2/s$for the 25% w/w PVP concentration.

Temperature control was achieved by equilibration with ice-water. The Phantom was scanned using the same acquisition protocols designed for use in the patient (Table 1).

Imaging equipment undergoing initial assessment consisted of sixteen scanners in total, consisting six different models of scanner from three manufacturers. Three of the scanners had a main field strength of 3T the remaining scanners were 1.5 T. Once the trial was open patient recruitment occurred at eight separate imaging centers in five countries and used ten separate scanners of which two had a field strength of 3T.

ADC maps were calculated on a pixel-by-pixel basis for the phantom scans. Automated regions of interest were placed over each of the five cylinders using a spatial template based on the phantom design (supplementary figure S1). Regions of interest were generated based on the center of each cylinder including 75% of the cylinder volume to avoid artifact adjacent to the wall of the cylinder. Measurements were taken from three adjacent slices of each cylinder and averaged. Region of interest placement was manually confirmed, and a number of quality control metrics are calculated. These included 1) error in mean ADC based on comparison with known value in the phantom and 2) fitting error of ADC based on mean residual values. From these values the correlation between mean measured ADC and

expected values were calculated and expected percentage difference from the known mean ADC value was calculated for each cylinder using the known phantom values. Similar values were calculated from ADC images and the correlation between known and expected percentage difference values were compared using Pearson's correlation coefficient.

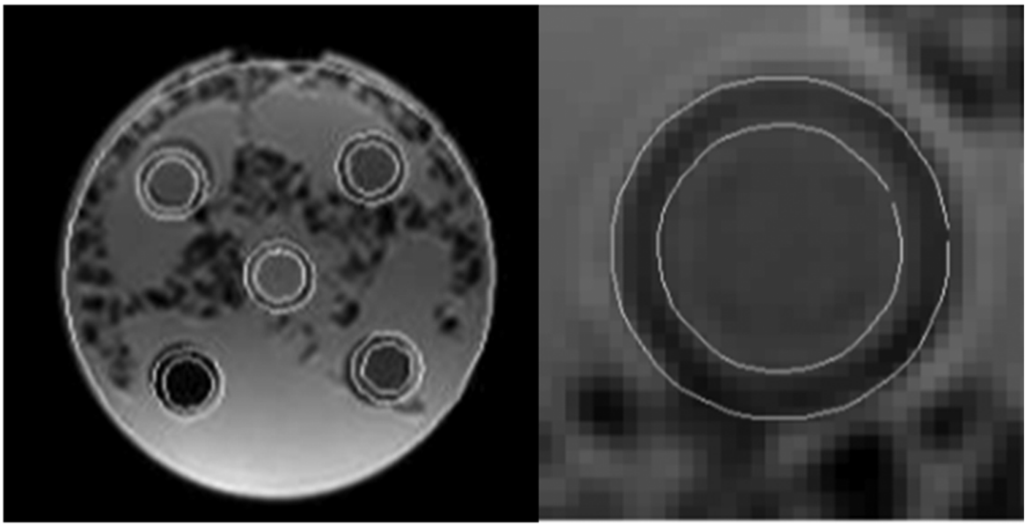

Figure S1: *ADC map of the phantom (left) and example of region-of-interest (ROI) placement (right).*

Phantom quality control results

Supplementary figure S2A shows the percentage difference between the expected and measured mean ADC values from the baseline phantom scans. The majority of scanners (13/16) showed less than 5% deviation. In three cases larger errors are identified. In two of these the errors were identified as failure to adequately wait for temperature equilibration of the phantom or use of inadequate amounts of ice. In these cases, repeat scanning placed the measurements within the 5% threshold (see stars which represent repeat scans at one example site). In one case the error was above 5% without clear cause and this center was excluded from the study. Supplementary figure S2B shows the mean percentage fitting error, averaged over all cylinders in the Phantom for all test scans from the remaining 15 the scanners at baseline. Fitting error was below 15% in all but three scans which represented

scans where inappropriate use of the Phantom had been identified in the previous stage. Supplementary figure S2C shows the correlation coefficients between actual and measured ADC values for each scanner, correlation coefficients were above 0.85 in all but four cases which again represented inappropriate use of the Phantom. Supplementary figure S2D is an example quality control plot showing the correlation between expected and measured proportional change from the mean value for each of the five cylinders in the Phantom. The left-hand image shows a typical control plot from a machine with high conformance between measured and predicted ADC values. The right-hand figure shows a plot from the machine that was excluded from the study.

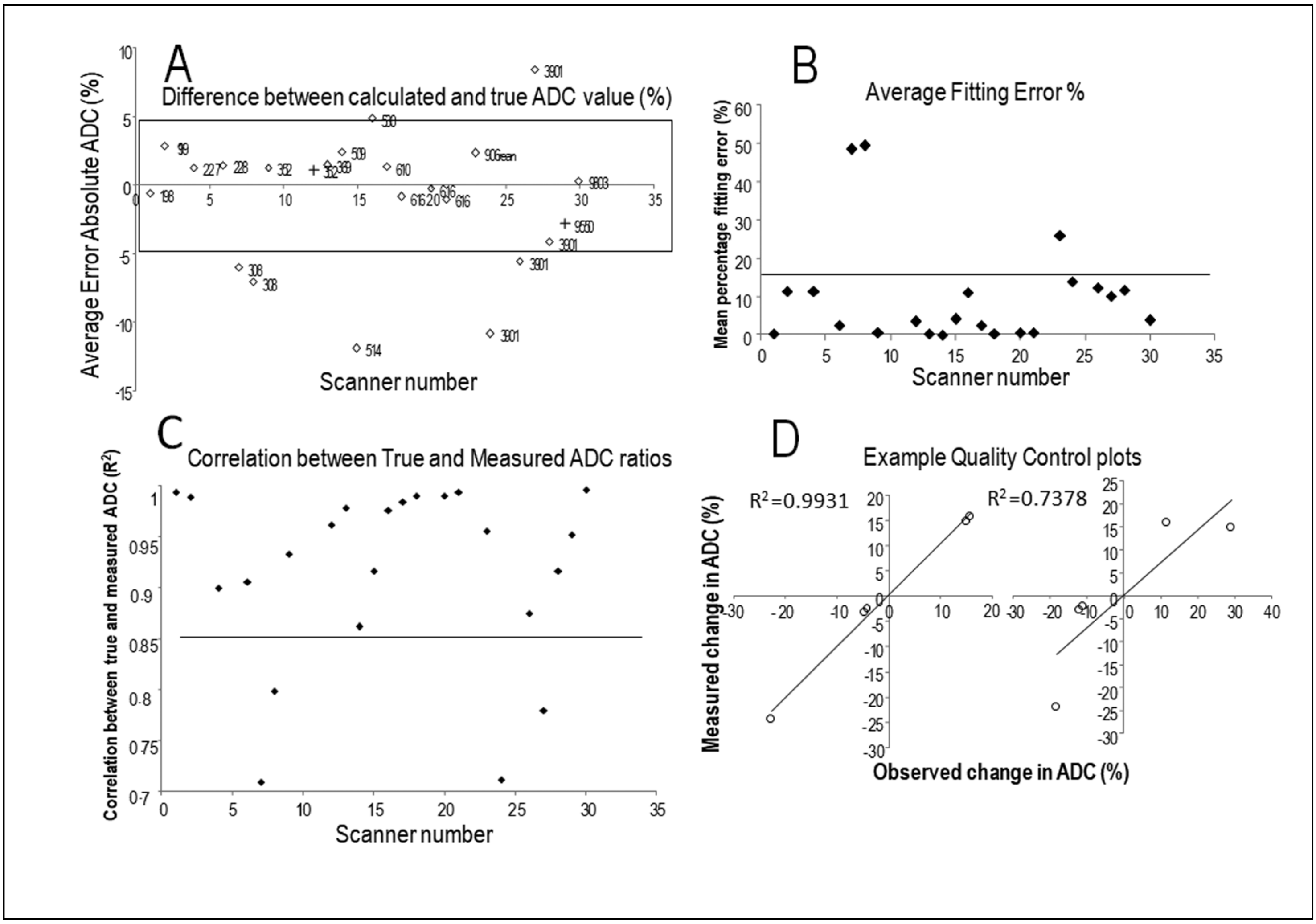

Figure S2: ADC measurements averaged over all cylinders in the phantom for each of the scanners at baseline showing A) percentage difference between calculated and true ADC values; B0 Mean percentage fitting error; C) correlation between measured and true ADC values. D shows example quality control plots of the correlation between expected and measured proportional change from the mean value for each of the 5 cylinders of the phantom in a machine with high conformance (left) and from the one whichh was excluded from the study (right).
